# Overexpression of the *Escherichia coli* TolQ protein leads to a null-FtsN-like division phenotype

**DOI:** 10.1002/mbo3.101

**Published:** 2013-07-02

**Authors:** Mary A Teleha, Adam C Miller, Ray A Larsen

**Affiliations:** 1 Department of Biological Sciences, Bowling Green State UniversityBowling Green, Ohio, 43403; 2 Division of Science and Math, Lorain County Community CollegeElyria, Ohio, 44035

**Keywords:** Cell division, *Escherichia coli*, FtsN, TolQ

## Abstract

Mutations involving the Tol-Pal complex of *Escherichia coli* result in a subtle phenotype in which cells chain when grown under low-salt conditions. Here, the nonpolar deletion of individual genes encoding the cytoplasmic membrane-associated components of the complex (TolQ, TolR, TolA) produced a similar phenotype. Surprisingly, the overexpression of one of these proteins, TolQ, resulted in a much more overt phenotype in which cells occurred as elongated rods coupled in long chains when grown under normal salt conditions. Neither TolR nor TolA overexpression produced a phenotype, nor was the presence of either protein required for the TolQ-dependent phenotype. Consistent with their native membrane topology, the amino-terminal domain of TolQ specifically associated in vivo with the periplasmic domain of FtsN in a cytoplasm-based two-hybrid analysis. Further, the concomitant overexpression of FtsN rescued the TolQ-dependent phenotype, suggesting a model wherein the overexpression of TolQ sequesters FtsN, depleting this essential protein from the divisome during Gram-negative cell division. The role of the Tol-Pal system in division is discussed.

Over-expression of the cytoplasmic membrane protein TolQ resulted in a division phenotype similar to that seen in cells depleted for FtsN. Two hybrid analysis suggested that TolQ and FtsN physically interact through domains that localize in the periplasmic space; while the concurrent over-expression of FtsN alleviated the TolQ over-expression phenotype. Together these results suggest a model wherein over-expressed TolQ sequesters FtsN, disrupting normal cell division.

## Introduction

The Tol system is a set of proteins that ostensibly function to couple cytoplasmic membrane (CM)-derived energy to outer membrane (OM) processes in the Gram-negative envelope (Cascales et al. 2000; Germon et al. 2001). These proteins are encoded from the *tol-pal* gene cluster, which is conserved among many Gram-negative bacteria with products sharing similarities in both sequence (Sturgis 2001) and, where examined, function (Webster 1991; Dennis et al. 1996; Heilpern and Waldor 2000; Prouty et al. 2002; Llamas et al. 2003). While the specific physiological role of the Tol system remains unclear, numerous phenotypes have been observed in Tol system mutants. Initially identified as conferring tolerance to certain colicins (Nagel de Zwaig and Luria 1967) and later filamentous phage (Sun and Webster 1986), *tol* mutants display a variety of traits consistent with disruption of OM integrity. These include formation of mucoid colonies, hypersensitivity to deoxycholate and high molecular mass antibiotics (Bernstein et al. 1972), leakage of periplasmic contents (Lazzaroni and Portalier 1981), and shedding of OM-derived vesicles (Bernadac et al. 1998). Other studies have suggested a potential role in the expression of O-specific lipopolysaccharides known to participate in OM integrity (Gaspar et al. 2000; Vines et al. 2005).

The *tol-pal* gene cluster consists of seven open reading frames, five of which encode proteins with established roles in the Tol system (Vianney et al. 1996). Two of these proteins, TolQ and TolR, are components of a heteromultimeric CM protein complex that appears to couple a third protein, TolA, to the electrochemical gradient of the CM (Cascales et al. 2000, 2001; Germon et al. 2001). TolA protein spans the periplasmic space to interact with a number of OM-associated proteins, including two *tol-pal* gene cluster products, TolB and Pal (Isnard et al. 1994; Cascales et al. 2002). The Tol protein complex, mediated through Pal, interacts with two non-*tol*-encoded proteins, OmpA and the major lipoprotein Lpp. Two additional genes, *ybgC* and *ybgF,* encode proteins whose contributions to the Tol system remain unclear (Cascales et al. 2002). The *ybgC* gene product localizes to the cytoplasm, where it functions as a thioesterase (Zhuang et al. 2002). The function of the periplasmically localized YbgF protein is unclear; however genetic and physical evidence suggests that it does interact with other proteins of the Tol system (Walburger et al. 2002; Krachler et al. 2010).

While maintenance of OM integrity is the most often cited role for the Tol system, indirect evidence has implicated this protein complex in the cell division process. Meury and Devilliers (1999) observed impaired cell division patterns in *Escherichia coli tolA* mutants when grown under conditions of either low osmolarity or high ionic strength. This distinct morphological phenotype, consisting of filamenting or chaining cells, was subsequently observed in *tol* mutants of *Vibrio cholerae* (Heilpern and Waldor 2000), *Pseudomonas putida* (Llamas et al. 2000), and *Erwinia chrysanthemi* (Dubuisson et al. 2005). Gerding et al. (2007) found that Tol mutants experience delayed OM invagination and contain OM blebs at constriction sites and cell poles. In that study, chimeras consisting of each of the five Tol proteins translationally fused with either green (GFP) or red (RFP) fluorescent protein appeared to localize to constriction sites during cell division. This localization did not occur when any of the Tol-GFP fusion proteins were expressed in cells depleted of the divisome protein FtsN. These data led the authors to suggest a model where Tol system components are recruited by FtsN to cell constriction sites where they then couple the OM to the divisome to coordinate division of the OM with septum formation and cell division (Gerding et al. 2007). It was subsequently observed that in the absence of FtsN, an FtsA suppressor mutation could support the localization of TolA-GFP to the divisome, indicating that the role of FtsN in this process is probably indirect (Bernard et al. 2007).

In previous studies, we had noted that even moderate overexpression of one particular Tol system component; TolQ, hindered the growth of *E. coli* strains in both fluid (Brinkman 2007) and solid phase cultures (Brinkman and Larsen 2008). Subsequently we observed that TolQ overexpression resulted in cells with a smooth, highly elongated appearance, a phenotype similar to that previously observed in FtsN-depleted cells (Dai et al. 1993). In this study, we document this TolQ-specific overexpression phenotype and provide genetic and biochemical evidence for direct interactions between TolQ and the divisome protein FtsN.

## Methods

### Media

Bacterial strains were maintained on Luria-Bertani (LB) agar (Miller 1972). Plasmid-bearing strains were maintained on LB agar supplemented with 100 μg mL^−1^ ampicillin, and/or 34 μg mL^−1^ chloramphenicol as necessary. For low osmotic assays, cells were grown in a modified LB broth, made without the normal addition of 1.0% (w/v) NaCl. For all other assays cells were grown in standard LB broth, supplemented with antibiotics as necessary, and l-arabinose as indicated. Colicin sensitivity assays were performed using cells suspended in T-top on T-plates (Miller 1972) as previously described (Larsen et al. 2003), with both the T-top and T-plates supplemented with antibiotics as necessary and l-arabinose as indicated. Two-hybrid analyses were performed on a variety of M9-based minimal selective media made as described in the BacterioMatch Two-Hybrid Instruction Manual (Stratagene: Agilent Technologies Inc., La Jolla, CA). These included: His-dropout Broth (1× M9 minimal salts containing 1× His-dropout supplement amino acids (Clontech Laboratories, Inc. Mountain View, CA), 0.4% (w/v) glucose, 200 μmol/L adenine HCl, 1 mmol/L MgSO_4_, 1 mmol/L Thiamine HCl, 100 μmol/L CaCl_2_, 50 μmol/L IPTG, and 10 μmol/L ZnSO_4_); nonselective agar (His-dropout broth, 1.5% (w/v) agar, chloramphenicol 25 μg mL^−1^ and tetracycline 12.5 μg mL^−1^); selective screening agar (nonselective agar with 5 mmol/L 3-amino-1,2,4 triazole); and dual selective screening agar (selective screening agar with 12.5 μg mL^−1^ streptomycin).

### Strains

Bacterial strains and plasmids are summarized in Table 1. The *E. coli* K12 strain W3110 (Hill and Harnish 1981) was used in this study as the wild type. The W3110 derivatives RA1027, RA1028, and RA1038 carried precise, complete deletions of the *tolQ*,* tolR*, and *tolA* genes, respectively. The construction of RA1038 was previously described (Weitzel and Larsen 2008). In the present study, deletions of *tolQ* and *tolR* were similarly created using the λ red recombination technique (Datsenko and Wanner 2000) to replace the predicted open reading frame for each gene with a “scar” region containing a stop codon and a ribosome-binding site to minimize polar effects on downstream genes. Tol phenotypes of deletion mutants and their complementation by plasmids (see below) were confirmed by sensitivities to deoxycholate and group A colicins (data not shown) as previously described (Brinkman and Larsen 2008; Weitzel and Larsen 2008). For two-hybrid analysis the “BacterioMatch II two hybrid system reporter” strain (*Δ*(*mcrA*)*183 Δ*(*mcrCB-hsdSMR-mrr*)*173 endA1 hisB supE44 thi-1 recA1 gyrA96 relA1 lac* [*F′ lacIq HIS3 aadA Km*^*r*^]) of *E. coli* K12 was purchased (Stratagene; Agilent Technologies Inc., La Jolla, CA). The *Enterobacter amnigenus* strain ATCC51816 and *Cronobacter muytjensii* strain ATCC51329 were obtained from the American Type Culture Collection.

**Table:** Bacteria and plasmids

Strains	Relevant characteristics^1^	Source
*Escherichia coli*		
W3110	F^−^ IN(*rrnD-rrnE*)1	Hill and Harnish (1981)
RA1027	W3110-Δ*tolQ*	Present study
RA1028	W3110-Δ*tolR*	Present study
RA1038	W3110-Δ*tolA*	Weitzel and Larsen (2008)
BacterioMatch II reporter	*hisB lac* [*F′ lacIq HIS3 aadA Km*^*r*^]	Stratagene^2^
*Enterobacter amnigenus*		
(ATCC51816)	Clinical isolate	ATCC^3^
*Cronobacter muytjensii*		
(ATCC51329)	Clinical isolate	ATCC^3^
Plasmids		
pBAD18-Cm	*araBAD* promoter, AraC, cm^r^	Guzman et al. (1995)
pBAD24	*araBAD* promoter, AraC, amp^r^	Guzman et al. (1995)
pKP315	pBAD24 encoding TonB	Larsen et al. (1999)
pKP660	pBAD24 encoding ExbBD	Ollis and Postle (2012)
pBT	lambda cI fusion site, cm^r^	Stratagene
pBT-LGF2	lambda cI-LGF2 fusion, cm^r^	Stratagene
pTRG	RNAPα fusion site, tet^r^	Stratagene
pTRG-Gal11^P^	RNAPα-Gal11^P^ fusion, tet^r^	Stratagene
pRA002	pBAD24 encoding TolR	Brinkman and Larsen (2008)
pRA003	pBAD24 encoding TolQR	Brinkman and Larsen (2008)
pRA004	pBAD24 encoding TolA	Weitzel and Larsen (2008)
pRA031	pBAD24 encoding TolQ	Present study
pMT001	pBAD24 encoding FtsN	Present study
pMT002	pBAD18-Cm encoding TolQ	Present study
pMT005	pBT lambda cI – TolQ(1-19)	Present study
pMT006	pBT lambda cI – TolQ(39-135)	Present study
pMT007	pBT lambda cI – TolQ(157-174)	Present study
pMT008	pBT lambda cI – TolQ(194-230)	Present study
pMT009	pTRG RNAPα – FtsN(1-33)	Present study
pMT010	pTRG RNAPα – FtsN(54-243)	Present study
pMT011	pTRG RNAPα – FtsN(54-319)	Present study

1 The specific nature of the deletions (∆) is as follows: The *∆tolQ* deletion removed the predicted *tolQ* codons 1-230 and the termination codon; the *∆tolR* deletion removed the predicted *tolR* codons 1-142 and the termination codon; the *∆tolA* deletion removed the predicted *tolA* codons 1-421, leaving the termination codon in place.

2 Purchased from Stratagene.

3 Purchased from the American Type Culture Collection (ATCC).

### Plasmids

Expression of specific Tol system proteins was achieved using plasmids derived from l-arabinose-regulated plasmids (Guzman et al. 1995) as previously described (Brinkman and Larsen 2008). For this study, the plasmid carrying only *tolQ* (pRA031) was constructed from pRA003 (carrying both *tolQ* and *tolR*) by removal of *tolR* with specific restriction, followed by ligation. This *tolQ* construct was then excised by restriction and ligated into the identically restricted pBAD18-Cm to create pMT002.

The *ftsN* gene was amplified by the polymerase chain reaction (PCR) from W3110 genomic DNA and inserted into pBAD24 at the NcoI and XbaI sites to create pMT001.

For two-hybrid analysis regions encoding the “bait” domains of TolQ were amplified by PCR and cloned into the plasmid pBT to generate in-frame fusions with a gene encoding the lambda cI protein. Four such fusions were constructed, using *tolQ* codons 1-19 (pMT005), 39-135 (pMT006), 157-174 (pMT007), and 194-230 (pMT008). Similarly, regions encoding “target” domains of FtsN were amplified and cloned into the plasmid pTRG to generate in-frame fusions with a gene encoding the α-subunit of RNA polymerase. Three such fusions were generated, using *ftsN* codons 1-33 (pMT009), 54-234 (pMT010), and 54-319 (pMT011).

The identities of all constructs were confirmed by DNA sequence analysis. Specific primers used for PCR are available on request.

### Microscopy

Fresh overnight cultures (grown at 37°C with agitation in LB broth supplemented with antibiotics as necessary) were diluted 1:200 into 5 mL aliquots of fresh LB broth, supplemented with antibiotics as necessary and l-arabinose as indicated. In preliminary studies cultures were grown by shaking at 37°C, with cells harvested as indicated with a bacteriological loop and heat-fixed onto clean glass slides. Fixed cells were stained with 0.6% (w/v) Safranin O in 20% (v/v) ethanol and then examined by light microscopy. Images of representative fields were digitally recorded under oil immersion on a Nikon H550S series compound light microscope using NIS Elements Documentation Software (Nikon Instruments Inc., Melville, NY).

### Immunoblot analysis

The relative levels of TolQ protein present in wild-type *E. coli* and in plasmid-bearing cells under l-arabinose induction were determined by immunoblot analysis. For the determination of relative induction levels (Fig. 3B), overnight cultures of W3110 carrying either pBAD24 or pRA031 were subcultured 1:200 in LB ampicillin 100 μg mL^−1^ supplemented with l-arabinose at 0.1%, 0.01%, 0.001%, 0.0001%, 0.00001%, or 0.0% (w/v) and incubated at 37°C with agitation. Cells were harvested in late exponential phase (A_550_ = 0.7, as determined with a Spectronic 20 spectrophotometer (Thermo Scientific, Madison, WI) with a path length of 1.5 cm) and precipitated at 4°C with 10% trichloroacetic acid (TCA) to limit proteolysis (Abelson et al. 1990), rinsed with 1 mL 100 mmol/L Tris-HCl (pH 7.9), then suspended in 25 μL 1 mol/L Tris-HCl, mixed with 25 μL 2× Laemmli sample buffer, incubated at 98°C for 5 min and stored at −20°C. Samples for dual overexpression studies were similarly processed, with cells cultivated in LB containing 34 μg mL^−1^ chloramphenicol and 100 μg mL^−1^ ampicillin and supplemented with l-arabinose at 0.0% or 0.1%, and harvested in stationary phase coincident with the harvesting of cells for microscopy (Fig. 6B). Samples were then resolved on a sodium dodecyl sulfate (SDS) 11% polyacrylamide gel (Laemmli 1970). Resolved proteins were transferred to a polyvinylidene fluoride membrane (Millipore Corp. Bedford, MA) and probed with a monospecific polyclonal rabbit antiserum raised against a synthetic peptide corresponding to TolQ residues 47-62 (generated by Pacific Immunology, San Diego, CA), and visualized using an anti-rabbit immunoglobulin horseradish peroxidase conjugate and enhanced chemiluminescence (ECL), as previously described (Larsen et al. 1993; Higgs et al. 1998).

### Bacterial two-hybrid analysis

Bait and target constructs were cotransformed into BacterioMatch^®^ II Screening Reporter Competent Cells according to the BacterioMatch^®^ II Two-Hybrid System protocol (Stratagene^®^, CA 92037). Cotransformation pairs consisted of pBT-TolQ^(1-19)^+pTRG-FtsN^(54-319)^, pBT-TolQ^(1-19)^+pTRG-FtsN^(54-243)^, pBT-TolQ^(157-174)^+pTRG-FtsN^(54-319)^, pBT-TolQ^(157-174)^+pTRG-FtsN^(54-243)^, pBT-TolQ^(39-135)^+pTRG-FtsN^(1-33)^, and pBT-TolQ^(194-230)^+pTRG-FtsN^(1-33)^. Cotransformants were plated on nonselective, selective, and dual-selective media, with dual transformants maintained on LB agar supplemented with tetracycline and chloramphenicol as described in the BacterioMatch Two-Hybrid Instruction Manual (Stratagene).

## Results

### The morphology of *∆tol* strains is altered when grown in low-salt medium

**Figure 1:**
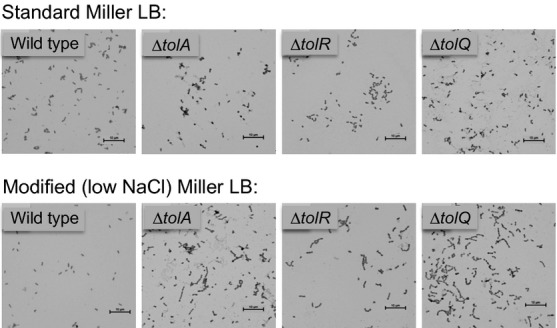
The morphology of *∆tol* strains is altered when grown in low salt medium. Stained preparations of W3110 (“wild type”) and the *∆tol* derivatives RA1038 (*ΔtolA*), RA1028 (*ΔtolR*), and RA1027 (*ΔtolQ*); grown for 24 h at 37°C with aeration in either normal Miller LB or modified Miller LB lacking added sodium chloride are shown. All panels are displayed at the same relative magnification, with a bar representing 10 μm provided in each panel for scale.

Distinct phenotypes for *∆tol* derivatives of W3110 were not evident by light microscopy for cells grown to stationary phase in standard LB at 37°C (Fig. 1). Conversely, when grown in low-salt LB, stationary phase *∆tol* strains occurred as short cocco-bacillary cells in chains, whereas the W3110 parent cells had a normal appearance as short, single rods (Fig. 1). Thus, the chaining phenotype previously noted specifically for *tolA* mutants grown in low salt (Meury and Devilliers 1999; Gerding et al. 2007) also occurred for *tolQ* and *tolR* mutants.

### The overexpression of TolQ uniquely results in cell filamentation

**Figure 2:**
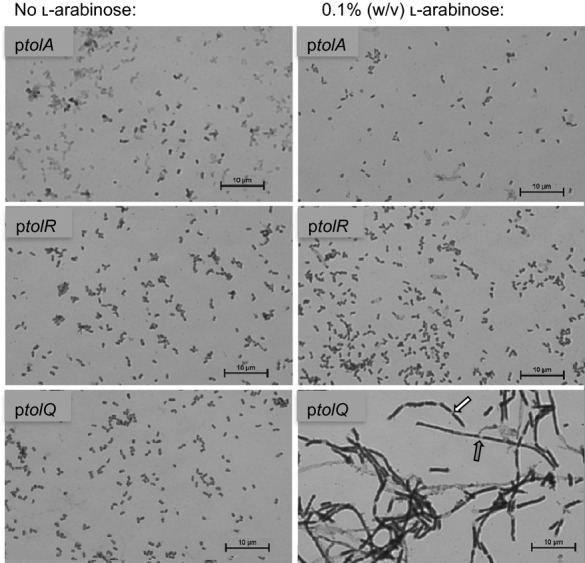
The overexpression of TolQ uniquely results in cell filamentation. Stained preparations of W3110 cells bearing plasmids carrying either the *tolA* (p*tolA*), *tolR* (p*tolR*), or *tolQ* (p*tolQ*) gene under the control of the pBAD promoter grown for 24 h at 37°C with aeration in Miller LB supplemented with 100 μg mL^−1^ ampicillin and either no l-arabinose or 0.1% (w/v) l-arabinose are shown. All panels are displayed at the same relative magnification, with a bar representing 10 μm provided in each panel for scale. The arrows in the lower right side panel highlight examples of possible septal formation.

The absence of individual Tol proteins resulted in a phenotype that suggested an impact on cell division when grown under nonstandard osmolarity conditions. Interestingly, the overexpression of these proteins resulted in distinctly different phenotypes. For TolA and TolR, overexpression had no evident impact on cell division when examined in stationary phase (Fig. 2). Surprisingly, TolQ overexpression resulted in a novel filamentation phenotype (Fig. 2, lower right panel). The majority of cells appeared as elongated rods growing in long chains, with regions suggestive of septum formation. This filamentation phenotype was not unique to stationary phase cells, as a similar morphology was evident in exponential phase cells in the first hours of growth following induction of *tolQ* expression (data not shown).

### The extent of cell filamentation corresponds to the level of TolQ expression

**Figure 3:**
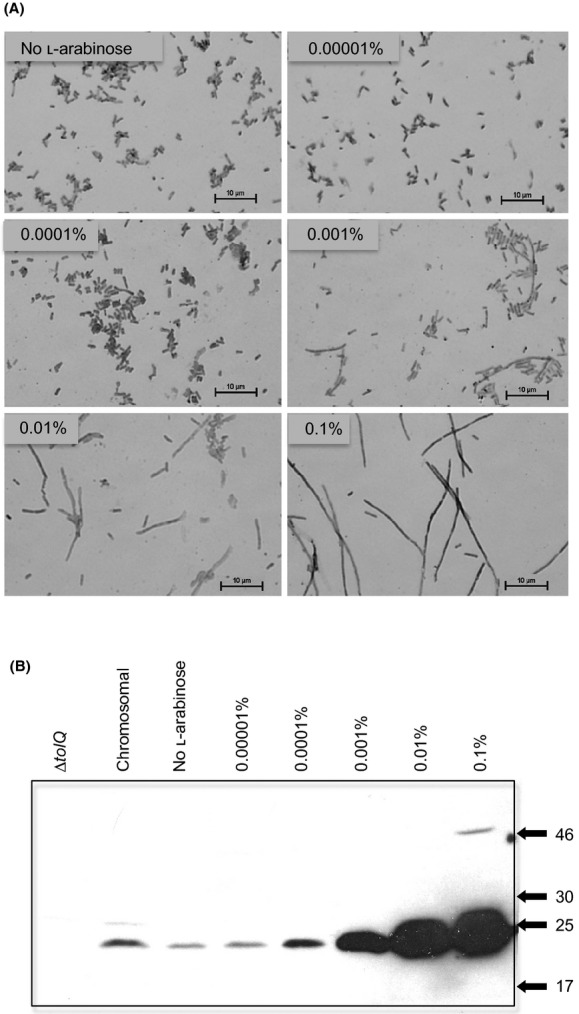
The extent of cell filamentation corresponds to TolQ expression levels. Figure 3A: Stained preparations of W3110 bearing a plasmid carrying the *tolQ* gene under the control of the pBAD promoter grown for 24 h at 37°C with aeration in Miller LB supplemented with 100 μg mL^−1^ ampicillin and either 0.0% or 10-fold increments of l-arabinose are shown. All panels are displayed at the same relative magnification, with a bar representing 10 μm provided in each panel for scale. Figure 3B: Immunoblot analysis of samples from cells grown to exponential phase in Miller LB supplemented with 100 μg mL^−1^ ampicillin and either 0.1% (w/v) l-arabinose for W3110 carrying the control plasmid pBAD24 (“chromosomal” and “*∆tolQ*”, respectively) or as indicated for W3110 carrying a pBAD24 derivative bearing the *tolQ* gene under l-arabinose control. Samples were resolved by sodium dodecyl sulfate polyacrylamide gel electrophoresis (SDS-PAGE) on an 11% polyacrylamide gel, transferred to a PVDF membrane and visualized by enhanced chemiluminescence using a monospecific anti-TolQ antiserum as described in Methods. The positions of molecular mass standards are indicated as kDa values at the right side of the developed blot. At higher levels of induction an additional, minor species of greater apparent molecular mass is detected by the anti-TolQ antiserum (0.1% lane).

If the filamentation phenotype occurs as a result of TolQ overexpression, the degree of filamentation should vary with level to which TolQ is overexpressed. Examination of cells carrying plasmids bearing the pBAD-regulated *tolQ* gene and grown to stationary phase in LB supplemented with increasing 10-fold concentrations of l-arabinose suggested this to be the case (Fig. 3A). In these experiments, cells grown with l-arabinose at levels of 0.0001% (w/v) or less were indistinguishable from those grown without l-arabinose supplementation, whereas filamentation is first evident in cells grown with 0.001% (w/v) l-arabinose and becomes increasingly apparent for cells grown in 0.01 and 0.1% (w/v) l-arabinose. Immunoblot analysis using a polyclonal monospecific TolQ antiserum allowed for the comparison of TolQ levels obtained by l-arabinose induction relative to the level of TolQ protein resulting from normal expression of the chromosomal *tolQ* gene (Fig. 3B). The relative levels of TolQ expression in these cells, assayed in late exponential growth, correlated directly with the filamentation phenotypes evident in stationary cells grown in the same concentrations of l-arabinose. Specifically, cells grown with l-arabinose concentrations that resulted in a TolQ level similar to chromosomally encoded levels did not show evidence of filamentation, with the filamentation phenotype first evident at an l-arabinose level that induces a moderate overexpression of TolQ (Fig. 3A, 0.001%) and becoming more extensive with l-arabinose levels that induce greater levels of TolQ protein.

### TolQ-induced cell filamentation occurs independent of TolR and TolA

**Figure 4:**
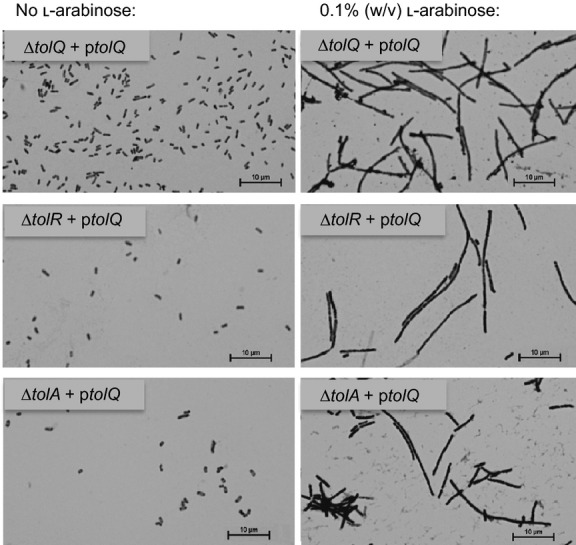
TolQ-induced cell filamentation occurs independent of TolR and TolA. Stained preparations of the W3110 *∆tol* derivatives RA1027 (*ΔtolQ*), RA1028 (*ΔtolR*), and RA1038 (*ΔtolA*) bearing a plasmid carrying the *tolQ* gene under the control of the pBAD promoter grown for 24 h at 37°C with aeration in Miller LB supplemented with 100 μg mL^−1^ ampicillin and either no l-arabinose or 0.1% (w/v) l-arabinose are shown. All panels are displayed at the same relative magnification, with a bar representing 10 μm provided in each panel for scale.

The above results suggested that neither the normal role of the CM Tol complex, nor a complete complex itself was involved in the filamentation phenotype. To test this possibility, TolQ protein was overexpressed in each of the *∆tol* strains, such that only in the case of the *∆tolQ* strain would all three components of the CM Tol complex be present (Fig. 4). The overexpression of TolQ resulted in the filamentation phenotype in all three *∆tol* strains, indicating that neither TolA nor TolR made essential contributions to the TolQ-dependent filamentation phenotype.

Because TolQ normally occurs in as part of a CM protein complex with TolR, the possibility that filamentation was a response to a stoichiometric imbalance between TolQ and Tol R was examined. Overexpression of TolQ and TolR from an arabinose-regulated cloned operon similarly resulted in cell filamentation, with levels of TolQ achieved from the plasmid-borne *tolQR* operon slightly lower than those achieved from a plasmid encoded *tolQ* alone (Fig. S1).

TolQ and TolR are paralogues of ExbB and ExbD of the TonB system, with enough shared function that molecular crosstalk occurs between the systems (Braun 1989). Despite its structural and functional similarity to TolQ, overexpression of ExbB (with ExbD) to levels similar to those that result in extensive filamentation for TolQ did not alter the division phenotype of the cell (Fig. S1). This suggests filamentation is a very specific response to the overexpression of TolQ, involving protein interactions unique to TolQ.

### The TolQ amino-terminal domain interacts with the FtsN periplasmic domain

**Figure 5:**
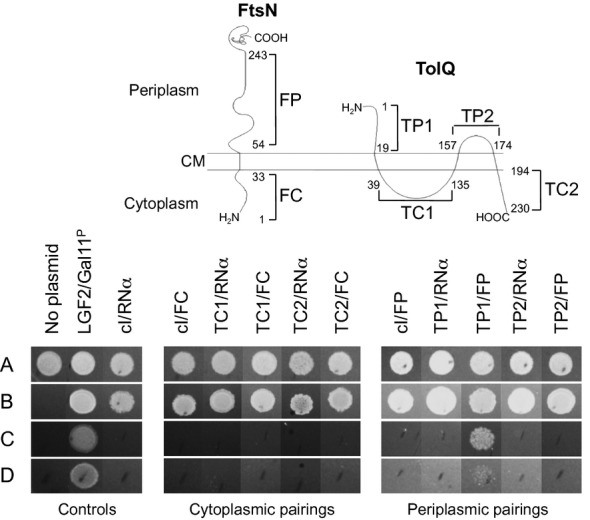
The TolQ amino-terminal domain interacts with the FtsN periplasmic domain. The potential for various regions of the TolQ and FtsN proteins to interact were tested by a two-hybrid analysis, using plasmids encoding the FtsN domains fused to RNαP2 and the TolQ domains fused to lambda cI. The individual regions considered for each protein are depicted by the diagram at the top, and identified by protein and topology relative to the cytoplasmic membrane. Thus, the cytoplasmically exposed FtsN residues 1-33 are termed “FC”, while the periplasmically localized FtsN residues 54-243 are termed “FP”. Similarly, the cytoplasmically exposed TolQ residues 39-135 and 194-230 are termed “TC1” and “TC2”, respectively, while the periplasmically localized TolQ residues 1-19 and 157-174 are termed “TP1” and TP2”, respectively. Indicator cells coexpressing various fusions and/or control products were plated on four different media and scored for growth at 24 h: “A”, LB medium; “B”, nonselective His dropout medium containing chloramphenicol 25 μg mL^−1^ and tetracycline 12.5 μg mL^−1^; “C”, selective screening medium comprised of nonselective agar supplemented with 5 mmol/L 3-amino-1,2,4 triazole; and “D”, dual selective screening medium comprised of selective screening agar supplemented with streptomycin 12.5 μg mL^−1^. Results are displayed as three sets: “Controls”, consisting of indicator cells with no plasmid, a positive control of two fusion proteins known to interact (LGF2/Gall11^P^), and a negative control pairing the lambda cI and RNαP2 encoding plasmids lacking fusion domains (cI/RNα); “Cytoplasmic pairings” consisting of indicator cells bearing combinations of plasmids encoding fusions of the cytoplasmic domains of FtsN and TolQ paired with each other or with cognate controls and; “Periplasmic pairings”, consisting of indicator cells bearing combinations of plasmids encoding fusions of the periplasmic domains of FtsN and TolQ paired with each other or with cognate controls.

The TolQ-dependent filamentation phenotype resembled the morphological phenotype previously reported for temperature-sensitive *ftsN* mutants grown under restrictive conditions (Dai et al. 1993). This suggested the possibility that the overexpression of TolQ somehow interfered with an FtsN-dependent process in cell division. The previous observation that FtsN appeared to play a role in recruiting GFP-Tol protein fusions to the divisome (Gerding et al. 2007) suggested a potential physical interaction between TolQ and FtsN. To address this possibility, “bait” domains of TolQ fused with the lambda cI protein and “target” domains of FtsN fused the α-subunit of RNA polymerase were paired for evaluation by bacterial two-hybrid analysis (Fig. 5). All strains and pairings grew on normal LB (Fig. 5 row A), and all but the “no plasmid control” grew on the chloramphenicol- and tetracycline-supplemented nonselective His dropout medium (Fig. 5, row B). The ability to grow under either selective (row C) or dual selective (row D) conditions was the criteria for putative interactions between paired domains of FtsN and TolQ, with the pBT-LGF2/pTRGGal11^P^pairing serving as a positive control for interaction, and the cI/RNα pairing providing a negative control. For the cytoplasmic domains, none of the pairings between FtsN and TolQ domains were productive (TC1/FC, TC2/FC), nor were the self-activation controls (cI/Fc, TC1/RNα, TC2RNα). For the periplasmic domains, a productive pairing occurred between the first periplasmic domain of TolQ and the periplasmic domain of FtsN, as indicated by growth on both selective and dual selective media (TP1/FP). Similarly, pairing of the entire periplasmic region of FtsN (residues 54-319) with the first periplasmic domain of TolQ also resulted in growth on both selective and dual selective media (data not shown). Productive interactions were not evident when the other TolQ periplasmic domain was paired with the FtsN periplasmic domain (TP2/FP), nor were the self-activation controls productive (cI/FP, TP1RNα, TP2/RNα).

### Overexpression of FtsN suppresses TolQ-induced cell filamentation

**Figure 6:**
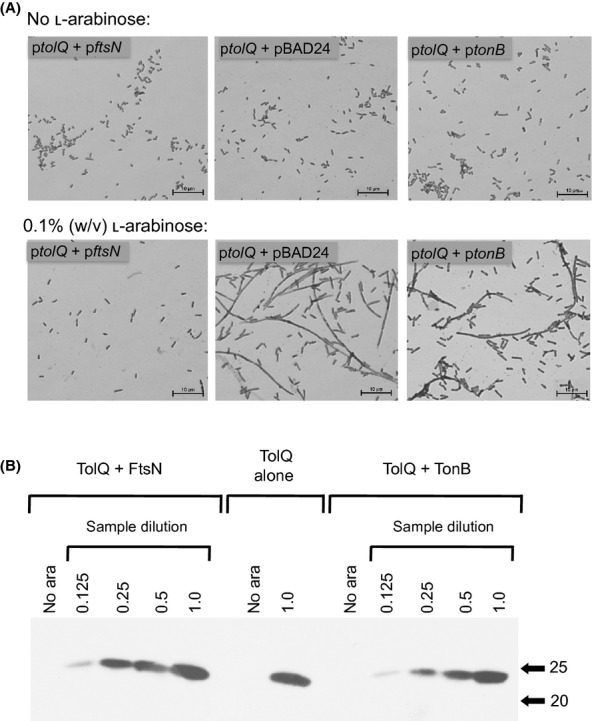
Overexpression of FtsN suppresses TolQ-induced cell filamentation. Figure 6A: Stained preparations of W3110 cells bearing a chloramphenicol-selected plasmid carrying the *tolQ* gene under the control of the pBAD promoter and paired with either an ampicillin-selected control plasmid (p*tolQ* + pBAD24), with a pBAD24 derivative carrying *ftsN* under the control of the pBAD promoter (p*tolQ* + p*ftsN*) or with a pBAD24 derivative carrying *tonB* under the control of the pBAD promoter (p*tolQ* + p*tonB*) grown for 24 h at 37°C with aeration in Miller LB supplemented with 100 μg mL^−1^ ampicillin and 34 μg mL^−1^ chloramphenicol are shown. Cultures were either not supplemented with l-arabinose or were supplemented with 0.1% (w/v) l-arabinose. Figure 6B: Immunoblot analysis of samples taken of the above cells at 24 h culture. A serial twofold dilution series was made for samples from cells coexpressing TolQ and either FtsN or TonB to allow for comparison of relative levels of TolQ protein, with “1.0” representing the undiluted sample and the other values representing the relative fraction thereof loaded onto the gel. Samples expressing TolQ alone and samples from cultures not supplemented with l-arabinose were loaded onto the gel without dilution. Samples were resolved by SDS-PAGE on an 11% polyacrylamide gel, transferred to a PVDF membrane and visualized by enhanced chemiluminescence using a monospecific anti-TolQ antiserum as described in Methods. The positions of molecular mass standards are indicated as kDa values at the right side of the developed blot.

The data from the two-hybrid analysis strongly support the likelihood of an in vivo interaction between TolQ and FtsN. In this setting, the overexpression of TolQ might result in a filamentation phenotype simply by binding to FtsN and preventing it from performing its normal function at the divisome. If so, then overproduction of FtsN should compensate for the loss of available FtsN due to TolQ sequestration and should alleviate the phenotype. To test this possibility, TolQ and FtsN were simultaneously overexpressed, using two l-arabinose-inducible pBAD expression vectors (Fig. 6A). As per previous experiments, the filamentation phenotype was observed when expression of the *tolQ* gene was induced with 0.1% (w/v) l-arabinose (Fig. 6A, p*tolQ* + pBAD24). Nevertheless, when paired with a plasmid bearing an l-arabinose-inducible *ftsN* gene, the filamentation phenotype was not observed in cultures grown with 0.1% (w/v) l-arabinose (Fig. 6A, p*tolQ* + p*ftsN*). This did not appear to result from an arabinose dilution effect as the control pairing of TolQ with TonB, a CM protein with a topology similar to that of FtsN still produced filamentous cells (Fig. 6A, p*tolQ* + p*tonB*). Despite the difference in filamentation phenotype, co-overexpression of TolQ with either FtsN or TonB resulted in similar levels of TolQ, indistinguishable from that of TolQ overexpressed alone (Fig. 6B).

### Overexpression of *E. coli* TolQ induces filamentation in other Gram-negative species

**Figure 7:**
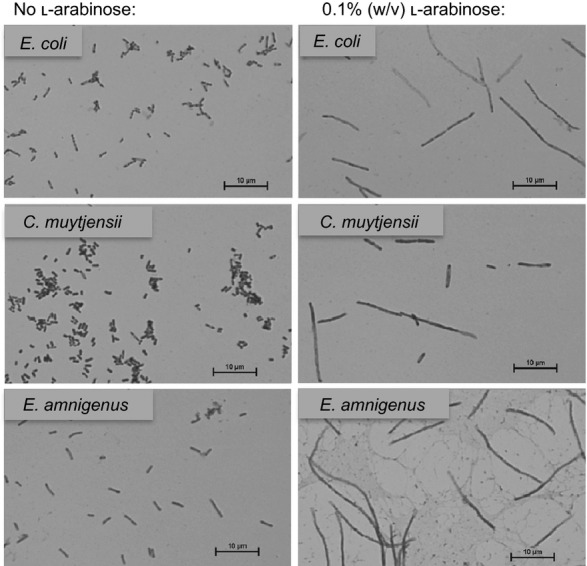
Overexpression of *Escherichia coli* TolQ induces filamentation in other Gram-negative species. Stained preparations of *E. coli*,* Cronobacter muytjensii*, and *Enterobacter amnigenus* strains bearing a plasmid carrying the *tolQ* gene under the control of the pBAD promoter grown for 24 h at 37°C with aeration in Miller LB supplemented with 100 μg mL^−1^ ampicillin and either no l-arabinose or 0.1% (w/v) l-arabinose are shown. All panels are displayed at the same relative magnification, with a bar representing 10 μm provided in each panel for scale.

The overexpression of TolQ protein appeared to interfere with some aspect of cell division in *E. coli* K12 strains. To determine if the TolQ-dependent filamentation phenotype was unique to this laboratory-adapted lineage or involved a general aspect of cell division in Gram-negative enterics, the l-arabinose-regulated *E. coli tolQ* gene was transformed into *E. amnigenus* and *C. muytjensii*. Under 0.1% (w/v) l-arabinose induction, both species, like *E. coli*, displayed a filamentation phenotype similar to that seen in *E. coli* when carrying the l-arabinose-regulated *E. coli tolQ* gene, but not when carrying the pBAD24 vector alone (Fig. 7).

## Discussion

First noted as a set of genes required for resistance to specific colicins (Nagel de Zwaig and Luria 1967), the *tol* genes and their products have been the subjects of much scrutiny. However, identification of their actual physiologic function has remained elusive. Evidence to date suggests that the Tol system couples the ion electrochemical gradient of the CM to the OM, with a variety of *tol* phenotypes reflecting energy dependence (Cascales et al. 2000; Germon et al. 2001; Vankemmelbeke et al. 2009). The pleiotropic phenotype displayed by *tol* mutants is suggestive of a role in the maintenance of OM integrity (Lloubés et al. 2001). Interestingly, certain *tol* system mutations impact the microscopic morphology of Gram-negative organisms, with cells grown under conditions of low osmolarity or high ionic strength displaying filamentous or chaining growth patterns (Meury and Devilliers 1999; Heilpern and Waldor 2000; Llamas et al. 2000; Dubuisson et al. 2005). The observation that Tol proteins fused to fluorescent proteins localized to division septa and that this trafficking was dependent upon the divisome protein FtsN led Gerding et al. (2007) to propose that the Tol proteins comprise a subcomplex of the division machinery, tethering the OM to the peptidoglycan corset during constriction of the divisome.

In the present study, we noted that *E. coli* strains individually deleted for *tolQ*,* tolR*, or *tolA* occurred as single cells when grown in standard LB medium, but, similar to previous observations with *tolA* strains (Meury and Devilliers 1999; Gerding et al. 2007) formed multiseptate cell chains when grown in LB lacking NaCl (Fig. 1). These observations indicate that the CM-associated components of the Tol system are not essential for normal resolution of division when cells are grown under standard osmotic conditions. However, when grown under low osmotic conditions, cells lacking any single component of the CM Tol complex displayed a chaining phenotype. In previous studies, the accumulation of either GFP-tagged TolA or TolQ at the nascent division septum occurred independent of the presence of other Tol proteins, whereas the trafficking of TolR to the divisome required the presence of TolQ (Gerding et al. 2007). Together these observations suggest the participation of the entire CM Tol protein complex in the division process, with central roles played by TolA and TolQ.

The division phenotype of strains lacking a component of the CM Tol complex was subtle, evident only when cells are grown under abnormal osmotic conditions. In contrast, the overexpression of TolQ resulted in a more obvious division phenotype in which cells appeared as elongated rods coupled in long chains (Fig. 2). Sites of apparent septal formation separated “individual” elongated rods. The spacing between these septal sites varied, likely reflecting the all-or-none nature of induction of the arabinose-inducible promoter used in this study (Siegele and Hu 1997). Consistent with this interpretation, the number of filaments relative to the number of “normal” cells increased in a dose-dependent manner (Fig. 3). Unlike the chaining phenotype of deletion mutants under low osmotic conditions, this filamentation phenotype was associated only with TolQ, with the overexpression of either TolA or TolR resulting in no evident division phenotype (Fig. 2). This suggested that the mechanism by which the overexpression of TolQ disrupted division was distinct from the mechanism by which the CM Tol complex contributes to cell division. Indeed, the filamentation phenotype occurred when TolQ was overexpressed regardless of the presence or absence of other components of the CM Tol complex (Fig. 4) indicating that this phenotype was not the result of an excess input of the normal contribution of the Tol system to cell division.

The phenotype of cells overexpressing TolQ is similar to that seen in FtsN-depleted cells, and it had been noted that FtsN was involved in recruiting Tol proteins to the divisome (Gerding et al. 2007); thus we hypothesized that high levels of TolQ might disrupt FtsN function. One possible mechanism for such a disruption would involve physical interactions between overexpressed TolQ protein and FtsN, limiting the availability of the latter to the divisome. To test this possibility we used a two-hybrid analysis strategy, first to evaluate the potential in vivo interaction between TolQ and FtsN and second to identify the specific domains involved in such an interaction (Fig. 5). These experiments suggested that TolQ and FtsN could interact in vivo; specifically the amino-terminal domain of TolQ (TolQ^(1-19)^) and the proximal periplasmic domain of FtsN (FtsN^(54-243)^) were identified as interacting regions. Significantly, this is the same FtsN domain previously identified as necessary to support proper cell division (Dai et al. 1996; Yang et al. 2004; Gerding et al. 2009). While these results are consistent with the topological partitioning of these membrane proteins, it was not possible to rule out additional interactions between the transmembrane domains of TolQ and FtsN by this approach.

If the TolQ-dependent filamentation phenotype did result from the sequestration of FtsN, the concomitant overexpression of FtsN should alleviate the phenotype. This was indeed the case (Fig. 6). Previously, it was shown that overexpressed TolQ localized to the membrane in a physiologically relevant conformation (Lewin and Webster 1996). Presumably, overexpressed FtsN is also trafficked to the CM appropriately. It should be noted that neither these nor the two-hybrid experiments exclude the possibility that interactions might occur between the cytoplasmic domain of TolQ and the periplasmic domain of nascent FtsN prior to its Sec-mediated insertion into the CM. However, such a mechanism would not be temporally consistent with the apparent recruitment of TolQ to the divisome.

The *tol-pal* gene cluster is conserved among Gram-negative species and the TolQ-TolR pair share homologues throughout the Eubacteria, indicating a long evolutionary history (Lazzaroni et al. 1999, 2002; Sturgis 2001). The homology of this system extends beyond sequence and protein structure similarities to functional ones, with Tol mutants in other species exhibiting common phenotypes tied to cell envelope integrity, OM maintenance (Sturgis 2001) and cell division (Meury and Devilliers 1999; Heilpern and Waldor 2000; Llamas et al. 2000; and Dubuisson et al. 2005). The homologous nature of this system would imply that similar roles for TolQ would be present in other Gram-negative bacteria. As expected, *E. coli* TolQ overexpressed in *E. amnigenus* and *C. muytjensii* resulted in a filamentation phenotype comparable to that observed in wild-type *E. coli* cells (Fig. 7), indicating that the TolQ overexpression phenotype is not an *E. coli*-specific phenomenon and is likely tied to the endogenous function of this protein. Because the Tol-Pal complex is widely conserved among Gram-negative bacteria and TolQ and TolR are the two most widespread proteins of the complex, sharing structural and functional homology with ExbB/ExbD and MotA/MotB (Eick-Helmerick and Braun 1989; Cascales et al. 2001), it is likely that the Tol-Pal complex originated with the appearance of Gram-negative bacteria. This protein complex, while its true role in the cell remains elusive, is clearly required for the maintenance of OM integrity (Lazzaroni et al. 1989), a role that can be understood as evidence that the Tol-Pal complex evolved alongside Gram-negatives and in particular, the Gram-negative OM.

Taken together, these data present an intriguing picture of a potential role for TolQ and by extension the Tol system in the process of cell division. The phenotype observed in cells overexpressing TolQ is distinct from that of Tol mutants grown in low-salt conditions. Overexpressed TolQ leads to the formation of long smooth filaments, a characteristic phenotype of *ftsN* mutants. The chaining phenotype observed in Tol mutants grown under low-salt conditions observed here and elsewhere (Meury and Devilliers 1999; Gerding et al. 2007) may reflect a response to osmotic stress by the absence of a functional Tol system. Cell filamentation has long been recognized as a stress response to unfavorable growth conditions such as nutrient limitation (Young 2006). Gerding et al. (2007) provided evidence that plasmid-expressed Tol proteins rescue this phenotype, while we found that excess TolQ results in a distinctly different division phenotype. TolQ-sequestered FtsN would potentially be unavailable in the cell to interact with another downstream essential cell division component to carry out its role in the process of cell division.

Although FtsN joins the septal ring near the end of its assembly, FtsN depletion leads to disassembly of the early divisome components of the proto-ring, indicating a specific role for FtsN in stabilization of the division ring. When restored to normal levels, FtsN back-recruits early divisome complexes (Rico et al. 2010) and a number of divisome components have been implicated in sharing a role in stabilizing the divisome, with functional overlap occurring among many (Geissler and Margolin 2005; Rico et al. 2010). This is evidenced by the ability of altered or highly expressed divisome components to compensate for the loss of others (Geissler and Margolin 2005; Bernard et al. 2007). Such findings support a model of divisome assembly and function that is more dynamic and cooperative than originally described. Further, interactions between divisome proteins and a growing number of nonessential proteins point to a more complex nature for the cell division process itself. It is reasonable to consider a dual role for the Tol-Pal complex in the cell. A role for the Tol system in OM maintenance has clearly been established in a number of Gram-negative species (Meury and Devilliers 1999; Heilpern and Waldor 2000; Llamas et al. 2000; Sturgis 2001; and Dubuisson et al. 2005). Much less is known about the role of the Tol system in cell division. Under standard conditions, Tol mutants continue to divide, ruling out an essential role for the Tol-Pal complex as a core component of the divisome. However, an accessory role for the Tol system in divisome stability is a very plausible physiological role for this system. The Tol-Pal system links the CM and OM, during both nondividing and dividing stages of the bacterial life cycle. During both stages, this link would have been advantageous throughout the evolution of the Gram-negatives. The ability of the Tol-Pal complex to interact with divisome components, in particular TolQ to interact with FtsN or FtsN-like proteins, would also have been advantageous, as such an interaction might provide additional stability to the divisome while physically linking the OM to the CM during cell division, facilitating a more efficient cell division process through cooperative binding. Evidence indicates the Tol proteins interact through their transmembrane domains to collectively perform energy-dependent functions in the cell (Germon et al. 2001) and that a necessary stoichiometric balance of Tol system components preserves this functionality (Guihard et al. 1994). Although the present data suggest an energized Tol system is unnecessary for generating the filamentation phenotype observed in cells overexpressing TolQ, it does not provide insight into the potential contribution of Tol system-derived energy in the division process as proposed by Gerding et al. (2007). While that study suggests that FtsN is normally involved in the recruitment of Tol proteins to the divisome, its role in this process appears to be indirect, as a suppressor mutation in FtsA can provide for TolA recruitment in an FtsN-depleted strain (Bernard et al. 2007). This suggests that the physical interaction between the periplasmic domains of TolQ and FtsN could occur following the recruitment of the Tol components to the divisome, with the overexpressed TolQ protein then diverting FtsN from its contributions to maintaining the divisome.

The details of Tol complex action at the division site remain to be determined. However, the results of this study support previous findings that the Tol-Pal complex, specifically TolQ, does indeed interact with the cell division machinery in *E. coli* and its relatives. We have identified the domains of interaction between TolQ and FtsN and provided evidence that this interaction is substantial enough to interfere with the cell division process when TolQ is present in excess. We have also shown that this disruption in cell division can be alleviated with simultaneous overexpression of FtsN. The mechanism by which the overexpression of TolQ and its interaction with FtsN disrupts the cell division process remain to be clarified, perhaps as the function of FtsN at the divisome becomes better understood and the specific role for the Tol-Pal complex during cell division is clarified.

## Supporting Information

**Figure S1**, TolQ-dependent cell filamentation is not mitigated by concurrent expression of TolR; whereas the TolQ paralogue ExbB does not induce filamentation when overexpressed. Figure S1A: Stained preparations of W3110 cells bearing plasmids carrying either the L-arabinoseregulated plasmid pBAD24 (pBAD24) or pBAD24 derivatives encoding *tolQ* (*ptolQ*), *tolQR* (*ptolR*), or exbBD (pexbBD) genes under the control of the pBAD promoter grown for 24 h at 37°C with aeration in Miller LB supplemented with 100 μg mL^-1^ ampicillin and either no L-arabinose or 0.1% (w/v) L-arabinose are shown. All panels are displayed at the same relative magnification, with a bar representing 10 lm provided in each panel for scale. Figure S1B: Immunoblot analysis of samples from cells used for stained preparations in Figure S1A (above). Samples were prepared by TCA precipitation as described in Methods, then resolved by SDS-PAGE on 11% polyacrylamide gels, transferred to a PVDF membrane, and visualized by enhanced chemiluminescence using a monospecific anti-TolQ antiserum and a polyspecific anti-ExbB antiserum as described in **Figure S1.** TolQ-dependent cell filamentation is not mitigated by concurrent expression of TolR; whereas the TolQ paralogue ExbB does not induce filamentation when overexpressed. Figure S1A: Stained preparations of W3110 cells bearing plasmids carrying either the l-arabinose-regulated plasmid pBAD24 (pBAD24) or pBAD24 derivatives encoding *tolQ* (p*tolQ*), *tolQR* (p*tolR*), or *exbBD* (p*exbBD*) genes under the control of the pBAD promoter grown for 24 h at 37°C with aeration in Miller LB supplemented with 100 μg mL^−1^ ampicillin and either no l-arabinose or 0.1% (w/v) l-arabinose are shown. All panels are displayed at the same relative magnification, with a bar representing 10 μm provided in each panel for scale. Figure S1B: Immunoblot analysis of samples from cells used for stained preparations in Figure S1A (above). Samples were prepared by TCA precipitation as described in. The positions of molecular mass standards are indicated as kDa values between the developed blot. Two nonspecific bands are evident in the samples probed with anti-ExbB (indicated by “*”), with the ExbB band evident at 25 kDa.
