# Supplementary figures and images for: Overexpression of the Escherichia coli TolQ protein leads to a null-FtsN-like division phenotype
Source: Microbiologyopen. 2013 Jul 2;2(4):618–32. doi: 10.1002/mbo3.101 (PMC3831626; doi:10.1002/mbo3.101)

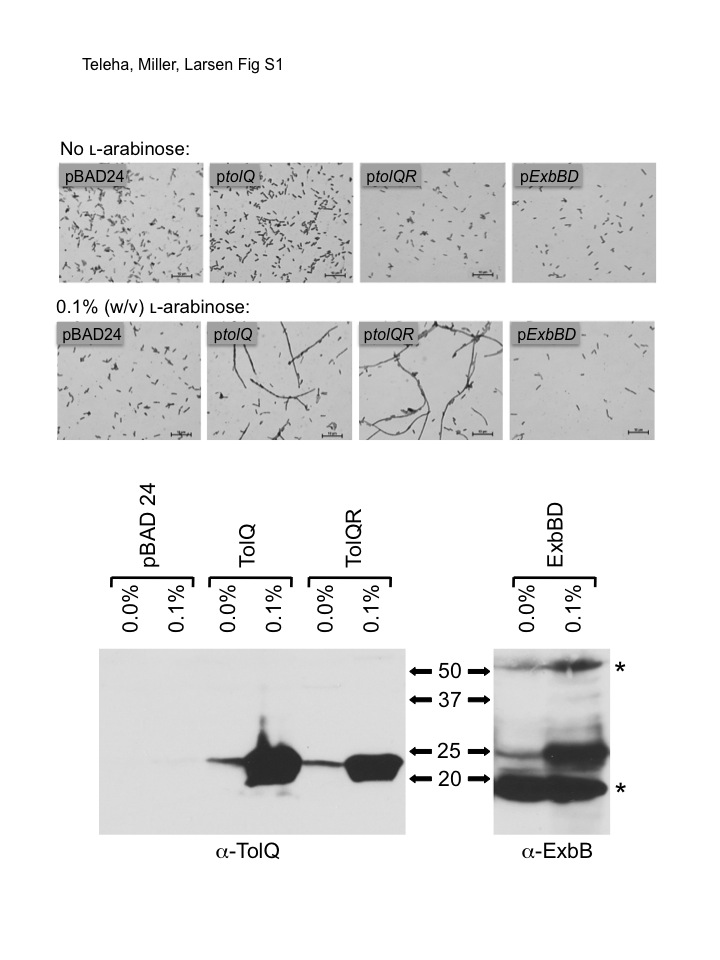

Supplement: Supplementary file 1 — Figure S1, TolQ-dependent cell filamentation is not mitigated by concurrent expression of TolR; whereas the TolQ paralogue ExbB does not induce filamentation when overexpressed. Figure S1A: Stained preparations of W3110 cells bearing plasmids carrying either the L-arabinoseregulated plasmid pBAD24 (pBAD24) or pBAD24 derivatives encoding tolQ (ptolQ), tolQR (ptolR), or exbBD (pexbBD) genes under the control of the pBAD promoter grown for 24 h at 37°C with aeration in Miller LB supplemented with 100 μg mL-1 ampicillin and either no L-arabinose or 0.1% (w/v) L-arabinose are shown. All panels are displayed at the same relative magnification, with a bar representing 10 lm provided in each panel for scale. Figure S1B: Immunoblot analysis of samples from cells used for stained preparations in Figure S1A (above). Samples were prepared by TCA precipitation as described in Methods, then resolved by SDS-PAGE on 11% polyacrylamide gels, transferred to a PVDF membrane, and visualized by enhanced chemiluminescence using a monospecific anti-TolQ antiserum and a polyspecific anti-ExbB antiserum as described in Figure S1. TolQ-dependent cell filamentation is not mitigated by concurrent expression of TolR; whereas the TolQ paralogue ExbB does not induce filamentation when overexpressed. Figure S1A: Stained preparations of W3110 cells bearing plasmids carrying either the l-arabinose-regulated plasmid pBAD24 (pBAD24) or pBAD24 derivatives encoding tolQ (ptolQ), tolQR (ptolR), or exbBD (pexbBD) genes under the control of the pBAD promoter grown for 24 h at 37°C with aeration in Miller LB supplemented with 100 μg mL−1 ampicillin and either no l-arabinose or 0.1% (w/v) l-arabinose are shown. All panels are displayed at the same relative magnification, with a bar representing 10 μm provided in each panel for scale. Figure S1B: Immunoblot analysis of samples from cells used for stained preparations in Figure S1A (above). Samples were prepared by TCA precipitation as described in. The positi [file mbo30002-0618-SD1.jpg]
